# Short sleep duration associated with increased risk for new-onset cardiovascular diseases in individuals with metabolic syndromes: Evidence from the China Health and Retirement Longitudinal Study

**DOI:** 10.3389/fcvm.2022.1010941

**Published:** 2022-11-07

**Authors:** Jiaxin Sun, Yizhou Chen, Yazhou Sun, Bo Yang, Jining Zhou

**Affiliations:** ^1^Department of Cardiology, Renmin Hospital of Wuhan University, Wuhan, China; ^2^Cardiovascular Research Institute, Wuhan, China; ^3^Hubei Key Laboratory of Cardiology, Wuhan, China; ^4^School of Computer Science, Wuhan University, Wuhan, China

**Keywords:** metabolic syndromes, short sleep duration, cardiovascular and cerebrovascular diseases, risk prediction, machine learning

## Abstract

To explore the impact and risk of short sleep duration (sleep duration < 6 h/night) on new-onset cardiovascular and cerebrovascular diseases (CVDs) in people with metabolic syndromes (Mets), this study used the 2011 baseline and 2015 follow-up data from the China Longitudinal Study of Health and Retirement (CHARLS) to conduct a prospective study of people aged ≥ 45 years in China. A total of 5,530 individuals without pre-existing CVDs in baseline were included. Mets were defined according to the harmonized criteria. We applied the Logistic Regression (LR), the Deep Neural Networks (DNN), and the Adaptive Boosting (AdaBoost), to evaluate the association between Mets components, short sleep, and the risk of new-onset CVDs, and the importance of multiple variates for new-onset CVDs. During the 4-year follow-up period, 512 individuals developed CVDs, and short sleep increased the risk of CVD in individuals with Mets. The odds ratio for prevalent CVD in Mets with short sleep group was 3.73 (95%CI 2.95–4.71; *P* < 0.001) compared to the normal group, and 1.99 (95% CI 1.58–2.51; *P* < 0.001) compared to the Mets without short sleep group. The DNN method reached the highest precision of 92.24% and f1-score of 95.86%, and the Adaboost method reached the highest recall of 99.92%. Both DNN and Adaboost have better predictive performance than LR and revealed short sleep duration and components of Mets are all the strongest predictors of CVD onset.

## 1. Introduction

Cardiovascular and cerebrovascular diseases (CVDs), including ischemic heart disease, heart failure, stroke, and other several cardiac and vascular conditions, are one of the major causes of unnatural deaths globally and significantly threaten living standards (1, 2). CVD caused approximately 17.8 million deaths worldwide in 2017 (3). In this scenario, identifying accurately the risk factors for CVD is vital for its prevention. Existing evidence demonstrates that metabolic syndrome (Mets) is associated with a higher risk of all-cause and CVD mortality (4). Mets was defined as a compound of risk factors for CVD, including raised blood pressure and dyslipidemia (elevated total triglycerides and lowered high-density lipoprotein cholesterol), elevated fasting glucose, and central obesity (5). The reported prevalence of the Mets was 33.9% (31.0% in men and 36.8% in women), which indicates that Mets affect approximately 454 million adults in China (6). Given its enormous socioeconomic burden, it is imperative to identify additional modifiable lifestyle factors to decrease the incidence of Mets and CVD. Sleep is an essential factor in maintaining metabolic homeostasis (7). Growing epidemiological evidence indicated that chronic short sleep may disturb the metabolism and lead to adverse health outcomes, including hypertension, myocardial infarction (MI), stroke, coronary heart disease (CHD) (8, 9), diabetes mellitus (10), and impaired memory (11), these are all risk factors for the development of CVD. Although short sleep duration has been shown to be a behavioral factor adversely affecting public health, Depner et al. (12), bare studies investigated the risk of it on new-onset CVD in patients with Mets. Considering that the synergistic effect of short sleep duration and metabolic abnormalities may increase the risk of CVD, this study aims to explore this issue using data from the China Health and Retirement Longitudinal Study (CHARLS).

## 2. Methods

### 2.1. Data source and study population

This study was based on nationwide data derived from the CHARLS. CHARLS is an ongoing survey enrolling a nationally representative sample of the Chinese population aged 45 or older from 150 counties/districts and 450 villages/urban communities (13). It collected information on social demographics, socioeconomic status, anthropometric measurement, blood tests, self-reported nighttime sleep duration data, and health status and functioning. The detailed sampling design and questionnaire protocol has been described elsewhere^1^. This study was approved by the Biomedical Ethics Committee of Peking University (IRB00001052-11015), and all participants provided written informed consent. The baseline information was conducted in 2011, and the enrolled individuals were followed up every 2 years. This study analyzed the 2011 baseline and 2015 follow-up data of CHARLS.

**Figure 1:**
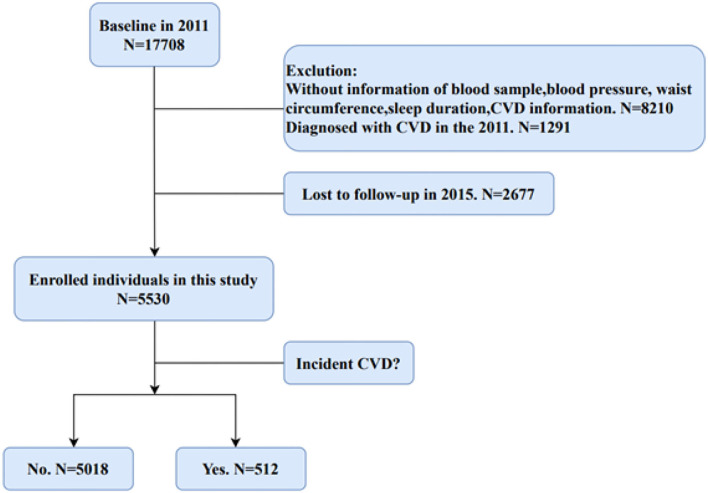
Flow chart of selection of eligible participants from the CHARLS.

Figure 1 shows the flow chart about the filtering process for eligible participants in our study. We selected participants without CVD in 2011 for the research from the 17,708 people who completed the baseline interviews. In addition, those participants without blood samples, information of sleep duration, blood pressure, and waist circumference were exclued. Finally, after 4 years of follow-up, 5,530 participants without CVD events in baseline were enrolled in this study.

### 2.2. Exposure features: Metabolic syndrome and short sleep duration

Fasting blood samples were stored at –80°C at the Chinese Center for Disease Control and Prevention in Beijing and measured at the Youanmen Clinical Laboratory of Capital Medical University (13). The following biomarkers were tested: fasting plasma glucose, glycosylated hemoglobin (HbA1c), total cholesterol (TC), high-density lipoprotein (HDL), low-density lipoprotein (LDL) cholesterols, triglycerides (TG), and uric acid (UA).

The blood pressure was measured by trained testers using an automatic blood pressure monitor (Omron HEM-7200 monitor) after the subject had rested in a seated position for approximately 30 min, and the cuff was wrapped around the subject's left upper arm for about 1–2 cm above the elbow fossa. The mean of three measurements was used. Hypertension was defined as mean systolic blood pressure ≥ 140 mmHg and/or diastolic blood pressure ≥ 90 mmHg (14), self-reported previous diagnosis of hypertension, or current use of drugs to control blood pressure (15). Waist circumference measurement required the participant to stand and breathe calmly. The examiner placed a measuring tape around the waist at the level of the participant's navel and measured waist circumference at the end of exhalation (16).

Based on the definitions in “Harmonizing the metabolic syndrome: a joint interim statement of the International Diabetes Federation Task Force on Epidemiology and Prevention; National Heart, Lung and Blood Institute; American Heart Association; World Heart Federation; International Atherosclerosis Society; and International Association for the Study of Obesity” (5), Metabolic syndromes was diagnosed when any three of the following indicators are met:

1. Elevated waist circumference (≥85 cm in men, ≥80 cm in women).
2. High blood pressure (Systolic ≥130 or diastolic ≥85 mmHg) or drug treatment for hypertension.
3. Low HDL cholesterol (<40 mg/dl in men and <50 mg/dl in women) or for drug treatment for low HDL.
4. High TG (≥150 mg/dl) or drug treatment for high TG.
5. Elevated fasting glucose (≥100 mg/dl or drug treatment for type 2 diabetes). Short sleep duration was defined as participants sleeping time < 6 h/day (8).

### 2.3. Outcome features: Self-reported CVD events

CVD events were obtained by participants who self-reporting that they had been diagnosed by a doctor with heart disease (including myocardial infarction, coronary artery disease, angina pectoris, congestive heart failure, and other heart conditions) or stroke. Participants with CVD events at baseline were excluded, and follow-up in 2015 with a new-onset CVD event was a positive outcome.

### 2.4. Covariates

The covariates included in our study were as follows: age, gender, body mass index (BMI), education (illiterate, non-illiterate), household registration (agriculture, non-agricultural), smoking behavior (smokers, non-smoker), drinking behavior (drinker, non-drinker), depression, and chronic kidney disease (CKD). Depressive symptoms were assessed using the ten items included in the Centre for Epidemiological Studies Depression Scale (CES-D) questionnaire (17). The total score ranges from 0 to 30. Depression was defined as a cut-off score of ≥ 10. Kidney function was evaluated by estimated glomerular filtration rate (eGFR). This study used the CKD-EPI equation to calculate eGFR (18). CKD (19) was defined as eGFR < 60mL/min/1.73 m^2^. BMI was calculated as Weight (kg)/ Height^2^ (m^2^).

### 2.5. Statistical analysis

Missing covariates are filled in with multiple interpolations to reduce selection bias and improve statistical power. Continuous variables were expressed as mean ± SD or median with interquartile ranges indicated; Categorical variables were expressed as numbers (percentages). Comparison of baseline characteristics of the metabolic syndrome by one-way ANOVA test (normal distribution) or chi-square test (categorical variables).

Multivariable logistic regression analysis was used to examine the associations between Metabolic syndrome (without short sleep and with short sleep) and new onset of CVD, and the odds ratio (ORs) with 95% confidence intervals (CIs) were calculated. Furthermore, we adjusted the model for age, gender, household registration, education, smoking behavior, drinking behavior, depression, and CKD in model 2. Moreover, we performed logistics regression analysis in different subgroups, including gender, age, smoking, drinking, depression, and CKD (with no adjustment for stratification variables in the corresponding models).

### 2.6. Machine learning method

This study exploited Logistic Regression (LR), the Deep Neural Networks (DNN) (20), and the Adaptive Boosting (AdaBoost) (21) to build predictive models which were designed to assess the association of 56 variables with the onset of CVD. In this work, we employed the random sampling method to generate the training set and test set, five-fold cross-validation to complete our experiments. Furthermore, the built-in weight-values in DNN and AdaBoost were used to assessed the importance of these variables, and higher variable importance indicates a more contributing role in the development of CVD.

The leading evaluation indicators for the three models (LR, DNN, and AdaBoost) are Precision, Recall, and F1-score. All statistical analyzes were performed using IBM SPSS Statistics 25.0 and Python 3.4.3. A bilateral *P* < 0.05 was considered statistically significant.

## 3. Results

### 3.1. Characteristics of the study population

The baseline characteristics of all adults in this study based on the Mets diagnosis and short sleep duration are shown in Table 1. A total of 5,530 participants in CHARLS were enrolled in this cohort, with 1,686 (30.5%) in the group of Mets without short sleep and 897 (16.2%) in the group of Mets with short sleep. The mean age of participants in 2011 was 58.25 years, and 54.2% were females. The participants in the group of Mets with short sleep were older (58.41 ± 8.94), more female (65.6%), and had the highest prevalence of CKD (14.8%), and depression (46.8%) than other groups. In addition, compared to normal group, both Mets without short sleep group and Mets with short sleep group had higher blood pressure, BMI, waist circumference, and blood biomarkers such as TC, TG, HDL, HbA1c, glucose, etc. (Table 1).

**Table 1:** Sample characteristics by metabolic syndromes amongst participants attending China Health and Retirement Longitudinal Study (CHARLS) (*N* = 5,530).

**Characteristics**	**Normal**	**Mets without short sleep**	**Mets with short sleep**	***P*** **-value**
	***N*** = **2,947(53.3%)**	***N*** = **1,686(30.5%)**	***N*** = **897(16.2%)**	
Age, SD, years	57.98(8.88)	58.36(8.99)	58.41(8.94)	<0.001
**Gender**				
Male, %	1,581(53.6)	645(38.3)	30934.4)	<0.001
Female, %	1,366(46.4)	1,041(61.7)	588(65.6)	<0.001
Illiterate, %	776(26.3)	462(27.4)	326(36.3)	<0.001
Agricultural domicile, %	2,598(88.2)	1,409(83.6)	769(85.7)	< 0.001
**Smoking**				
Smoker, %	1,306(44.3)	548(32.5)	262(29.2)	<0.001
Non-smokers, %	1,641(55.7)	1,138(67.5)	635(70.8)	<0.001
**Drinking**				
Drinker, %	1,103(37.4)	508(30.1)	650(27.5)	<0.001
Non-drinker, %	1,844(62.6)	1,178(69.9)	247(72.5)	<0.001
ADL/IADL difficulty, %	1,773(60.2)	1,029(61.0)	517(57.6)	0.238
**Physical activity**				
VPA, %	534(18.1)	252(14.6)	160(17.8)	0.84
MPA, %	772(26.2)	422(25.03)	244(27.2)	0.514
LPA, %	1,035(35.1)	567(33.63)	312(34.8)	0.164
CKD, %	268(9.1)	212(12.6)	133(14.8)	<0.001
Hypertension, %	679(23.0)	936(55.5)	502(56.0)	<0.001
Dyslipidemia, %	572(19.4)	1,206(71.5)	638(71.1)	<0.001
Elevated blood pressure, %	969(32.9)	1,220(72.4)	637(71.0)	<0.001
Elevated fasting glucose, %	1,168(39.6)	1,397(82.9)	717(79.9)	<0.001
Elevated triglycerides, %	223(7.6)	786(46.6)	415(46.3)	<0.001
Short sleep, %	879(29.8)	0(0.0)	897(100.0)	<0.001
Depression, %	1,444(49.0)	749(44.4)	420(46.8)	0.011
BMI, SD kg/m2	22.49(3.44)	24.91(3.67)	25.25(3.89)	<0.001
Waist circumference, SD cm	80.71(12.19)	88.81(10.69)	86.75(11.80)	<0.001
SBP, SD, mmHg	123.99(21.14)	135.85(21.01)	137.29(21.86)	<0.001
DBP, SD, mmHg	72.75(11.59)	79.02(11.99)	78.76(11.99)	<0.001
TC, SD, mg/dl	190.94(35.92)	196.13(40.46)	195.56(41.36)	<0.001
TG, SD, mg/dl	97.03(44.63)	174.18(151.19)	169.61(118.97)	<0.001
HDL, SD mg/dl	56.18(14.29)	45.90(14.98)	45.31(13.69)	<0.001
LDL, SD, mg/dl	117.10(32.04)	114.93(36.49)	114.97(38.04)	0.069
Glu, SD, mg/dl	101.84(25.94)	114.89(36.53)	117.53(39.78)	<0.001
HbA1c, SD, %	5.14(0.63)	5.36(0.90)	5.45(1.05)	<0.001
UA, SD, mg/dl	4.29(1.18)	4.48(1.27)	4.47(1.26)	<0.001
eGFR, SD, ml/min/1.73m2	83.05(15.59)	79.11(15.97)	77.44(15.95)	<0.001

### 3.2. Impact of metabolic syndromes with short sleep on CVD

Tables 2, 3 demonstrated the association between short sleep duration, Mets, and risk of new-onset CVD, in which Table 2 used the normal group as a reference and Table 3 used Mets with short sleep group as a reference. Both Mets and short sleep duration were directly associated with CVD incidence, and short sleep increased the risk of CVD in Mets. During the 4-year follow-up, A total of 165 (5.6%), 176 (10.4%), and 171 (19.1%) people in the group of normal, Mets without short sleep, and Mets with short sleep developed CVD, respectively (Table 2). Compared to the normal group, the odds ratios for incident CVD in fully adjusted multivariable logistics analysis (Model 2) were 1.88 (95% CI 1.50–2.35) and 3.73 (95% CI 2.95–4.71) in Mets without short sleep and Mets with short sleep group, respectively (Table 2). Furthermore, compared to the Mets without short sleep group, the new-onset CVD risk for Mets with short sleep was 2.00 (95% CI 1.59– 2.52) (Table 3).

**Table 2:** The association between short sleep duration, Mets, and risk of new-onset CVD, normal group used as a reference.

**Group (Incident CVD in 2011–2015)**	**Model 1**	**Model 2**
	**OR (95% CI)**	**OR (95% CI)**
Normal (*n* = 165, 5.6%)	1	1
Mets without short sleep (*n* = 176, 10.4%)	1.97 (1.58–2.45)	1.88 (1.50–2.35)
Mets with short sleep (*n* = 171, 19.1%)	3.97 (3.16–4.99)	3.75 (2.97–4.74)

Model 1: crude model.

Model 2: adjusted age, gender, household registration, education, smoke behavior, drink behavior, depression, and CKD.

**Table 3:** The association between short sleep duration, Mets, and risk of new-onset CVD, Mets with short sleep group used as a reference.

**Group (Incident CVD in 2011–2015)**	**Model 1**	**Model 2**
	**OR (95% CI)**	**OR (95% CI)**
Mets without short sleep (*n* = 176, 10.4%)	1	1
Mets with short sleep (*n* = 171, 19.1%)	2.02 (1.61–2.54)	2.00 (1.59–2.52)
Normal (*n* = 165, 5.6%)	0.51 (0.41–0.64)	0.53 (0.43–0.67)

**Figure 2:**
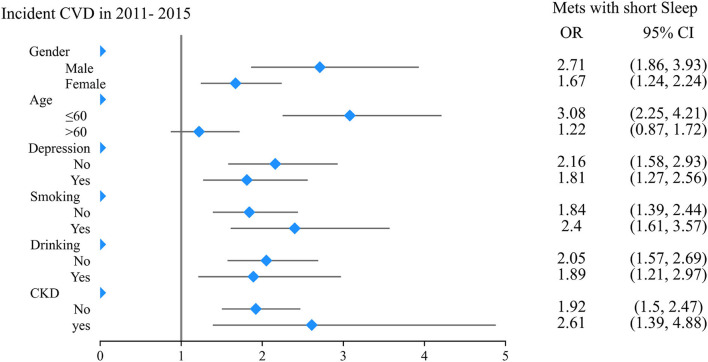
Forest plots of subgroup analysis on Mets with short sleep and risk of new-onset CVD.

Subgroup analysis showed consistent trends in the relationship between Mets, short sleep, and new-onset CVDs (Figure 2). In the subgroups of males, aged ≤ 60 years, smoking, and drinking, the odds ratio of new-onset CVD for people in Mets with short sleep group were 6.21 (95% CI 4.35–8.87), 5.55 (95% CI 4.06–7.58), 6.08 (95% CI 4.10–9.02), and 5.40 (95% CI 3.40–8.40), with normal group used as a reference (Table 4), which were significantly higher than people in Mets without short sleep group. Furthermore, when Mets without short sleep group was used as a reference, the risk of new-onset CVDs may increase by 2 to 3 folds for Mets with short sleep group. As show in the Table 5, such as 2.71 (95% CI 1.86–3.93), 3.08 (95% CI 2.25 4.21), 2.40 (95% CI 1.61–3.57), and 1.89 (95% CI 1.21–2.97) within above subgroups, respectively.

**Table 4:** The association between short sleep duration, Mets, and risk of new-onset CVD in subgroup analysis, normal group used as a reference.

**Model 2:**	**Normal**	**Mets without short sleep**	**Mets with short sleep**	***P*** **-value**
	**OR (95% CI)**	**OR1 (95% CI)**	**OR 2(95% CI)**	
**Incident CVD in 2011–2015**				
**Gender**				
Male	1	2.29 (1.62–3.28)	6.21 (4.35–8.87)	<0.001
Female	1	1.54 (1.15–2.06)	2.56 (1.89–3.48)	<0.001
**Age**				
≤ 60	1	1.80 (1.32–2.47)	5.55 (4.06–7.58)	<0.001
> 60	1	1.99 (1.44–2.77)	2.42 (1.69–3.42)	<0.001
**Depression**				
No	1	1.73 (1.28–2.34)	3.73 (2.73–5.11)	<0.001
Yes	1	2.08 (1.48–2.91)	3.75 (2.64–5.33)	<0.001
**Smoking**				
No	1	1.54 (1.16–2.03)	2.82 (1.12–3.77)	<0.001
Yes	1	2.53 (1.73–3.70)	6.08 (4.10–9.02)	<0.001
**Drinking**				
No	1	1.59 (1.22–2.07)	3.27 (2.49–4.28)	<0.001
Yes	1	2.85 (1.86–4.37)	5.40 (3.40–8.40)	<0.001
**CKD**				
No	1	1.93 (1.52–2.45)	1.49 (0.74–2.99)	<0.001
Yes	1	3.71 (2.89–4.77)	3.88 (1.98–7.60)	<0.001

**Table 5:** The association between short sleep duration, Mets, and risk of new-onset CVD in subgroup analysis, Mets with short sleep group used as a reference.

**Model 2:**	**Mets without short sleep**	**Mets with short sleep**	***P*** **-value**
	**OR (95% CI)**	**OR (95% CI)**	
**Incident CVD in 2011–2015**			
**Gender**			
Male	1	2.71 (1.86-3.93)	<0.001
Female	1	1.67 (1.24-2.24)	0.001
**Age**			
≤ 60	1	3.08 (2.25-4.21)	<0.001
> 60	1	1.22 (0.87-1.72)	0.258
**Depression**			
No	1	2.16 (1.58-2.93)	<0.001
Yes	1	1.81 (1.27-2.56)	0.001
**Smoking**				
No	1	1.84 (1.39-2.44)	<0.001
Yes	1	2.40 (1.61-3.57)	<0.001
**Drinking**			
No	1	2.05 (1.57-2.69)	<0.001
Yes	1	1.89 (1.21-2.97)	0.005
**CKD**			
No	1	1.92 (1.50-2.47)	<0.001
Yes	1	2.61 (1.39-4.88)	0.003

### 3.3. Experimental results of machine learning methods

**Figure 3:**
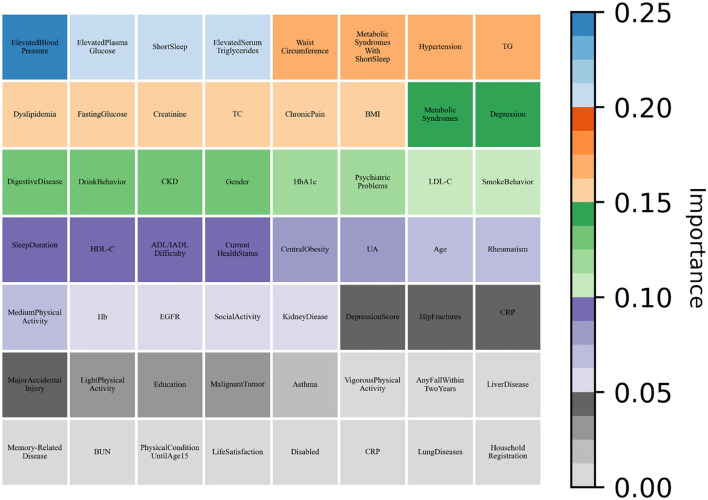
DNN evaluated the variable importance for the future incidence of CVD by weight values.

**Figure 4:**
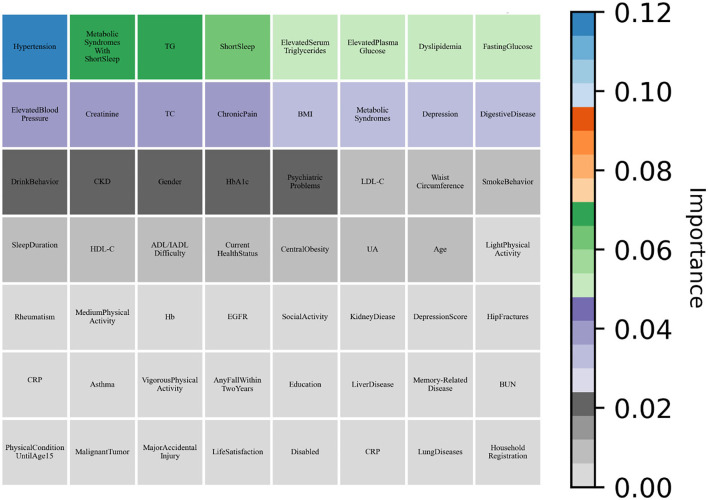
AdaBoost evaluated the variable importance for the future incidence of CVD by weight values.

As shown in Table 6, all machine learning methods exhibit excellent performance (the maximum value of each evaluation indicator is marked in bold) and achieved the average value for precision of 90.65%, recall of 99.28%, and f1-score of 94.77%. Notably, DNN performs best with precision of 92.24% and f1-score of 95.86%, AdaBoost reached the highest value of 99.92% in terms of recall. Finally, the variable importance was evaluated by built-in weights of DNN and AdaBoost and the results were summarized (Figures 3, 4), which showed that short sleep and components of Mets played essential roles in predicting CVD incidence.

**Table 6:** The performance of DNN, AdaBoost, and LR.

**Methods**	**Recall**	**Precision**	**F1-score**
DNN	99.76	**92.24**	**95.86**
AdaBoost	**99.92**	89.87	94.63
LR	98.15	89.84	93.81

Precision is the ratio of positive categories correctly predicted to all samples predicted to be positive. Recall is the ratio of correctly predicted positive categories to all actual positive samples. The F1-score takes into account both precision and recall. It is calculated by taking the summed average of the two metrics. The main evaluation indicators for machine learning are Precision, Recall and F1-score.

## 4. Discussion

In this nationally representative longitudinal study of middle and old aged population in China, three models of LR, DNN, and AdaBoost demonstrated short sleep duration is an independent risk factor for developing CVD, especially in Mets people. However, DNN, AdaBoost represent higher accuracy, compared to logistic regression model. Short sleep, elevation of blood pressure, blood glucose, triglyceride, waist circumference are valid predictor of new-onset CVD.

In terms of the association between sleep duration and physical ailments, many studies have shown that short-duration sleepers (< 6 h/night) increased risk of reporting hypertension (RR 3.59, 95% CI 1.58–8.17), and CKD (RR 1.63, 95% CI 1.28–2.08) compared to those with self-reported sleep of 7 h/night (22–24). A meta-analysis of 15 studies reported both short sleep duration (< 6 h/night) were associated with a greater risk of developing coronary heart disease (RR 1.48, 95% CI 1.22–1.80 and RR 1.38, 95% CI 1.15–1.66), stroke (RR 1.15, 95% CI 1.00– 1.31 and RR 1.65, 95% CI 1.45–1.87), compared to the reference category of 7–8 h of sleep (8). In addition, short-duration sleepers had an increased risk (HR 1.36, 95% CI 1.14–1.62) for cognitive impairments, including dementia compared to those without short sleep (11), which could be linked to the greater amyloid-beta burden and greater depressive symptoms, emphasizing the importance of maintaining adequate sleep (25). Furthermore, there have studies also reported a U-shaped relationship between nighttime sleep and CVD (26, 27). A cross-sectional analysis in the National Health Interview Survey showed increased risks of any CVD in short and long sleepers. After adjusting for known risk factors, the multivariate odds ratios of CVD for those sleep duration < 5 or > 9 h were 2.20 (95% CI 1.78, 2.71) and 1.57 (95% CI 1.31, 1.89), respectively (9).

In our study, the synergistic effect of short sleep duration and metabolic dysregulation associated with a doubling of CVD risk. The finding is in line with previous studies. Previous studies have shown that chronic metabolic dysfunction in insulin resistance and impaired glucose tolerance are substantial risk factors for CVD morbidity and mortality (10, 28). Sleep deprivation and circadian rhythm disturbances associated with metabolic dysregulation may lead to weight gain, obesity, and type 2 diabetes by increasing sympathetic nerve activity, disrupting energy balance, promoting inflammation, and impairing glucose tolerance and insulin sensitivity, causing cardiovascular damage (12). There have experiments suggested that sleep restriction seem to induce an increase in levels of interleukin-6 (IL-6) and C-reaction protein (CRP), which also indicate low-level systemic inflammation and play a role in metabolic disturbances (29, 30). Moreover, a sleep deprivation experiment that revealed diminished heart rate variability (HF-HRV) and high levels of plasma norepinephrine (NE) in people deprived of sleep for 26 h (31). The autonomic dysfunction in the form of decreased parasympathetic activity and increased sympathetic activity induced by sleep restriction is the risk factor for CVD, undoubtedly.

Sleep deprivation disturbs normal circadian rhythms, which in turn interferes with metabolism. The mammalian circadian rhythm is regulated by the suprachiasmatic nucleus (SCN) located in the hypothalamus, which followed the diurnal cycle and coordinated the release of neurotransmitters such as serotonin, norepinephrine, and melatonin. Short sleep could cause metabolic disorders by imbalanced the above hormones (32–34), and recent study has shown that lower levels of melatonin production are associated with a higher risk of developing type 2 diabetes. In addition, metabolic disturbances associated with sleep loss may also be mediated through gut dysbiosis and activation of the hypothalamic-pituitary-adrenal (HPA) axis (35). The microbiota is affected by circadian rhythms, stressors, diet, and exercise, sleep loss enhanced the translocation of viable bacteria from the intestine. Alterations in circadian variation in the composition and functionality of the gut microbiome have been identified as potential contributors to metabolic dysfunction during jet lag and shift work (36, 37). Furthermore, the interaction of the HPA axis and pro-inflammatory cytokines affect the sleep-arousal cycle. Hypercortisolism and hypercytokinemia play an important role in low sleep efficiency, fatigue, obesity, and metabolic dysregulation (38).

Numerous studies have observed that short sleep duration is associated with higher total energy intake and higher total fat intake as well as micronutrient inadequacy and vitamin D deficiency (39, 40). Furthermore, decreased leptin levels, elevated ghrelin levels and increased hunger and appetite also linked to short sleep (41). These nutritional and physiological hormones imbalances could make people obesity, metabolic disorder and type 2 diabetes in children and middle-aged and elderly people (42, 43). Two recent studies of adults with lifestyle restrictions leading to short sleep found improved insulin sensitivity after approximately 1 h of extended sleep per night In this study, people in Mets with short sleep group had the highest fasting blood glucose, glycated hemoglobin and BMI, which was consistent with previous results. Finally, short sleep may affect metabolic function through socio-psychological factors such as depression, psychological stress, low socioeconomic status, low physical activity and poor physical health (26), each of these psychosocial factors influences CVD risk. In summary, the synergistic effect of short sleep duration and metabolic abnormalities increases the risk of CVD.

This research highlights the need and importance of including short sleep in CVD risk prediction and assessment, especially in people with metabolic syndrome, sleep duration and quality should be monitored to prevent CVD. In this study, short sleep increased more than twice the risk of developing CVD (OR 2.61, 95% CI 1.39–4.88) than without short sleep, for people who had both chronic kidney diseaseand and Mets. If short sleep duration was neglected, the evaluation of developing CVD risk would be lowered. In addition, our findings may have important clinical implications for assessing sleep function in patients with CVD and initiating initiatives focused on improving sleep to reducing the risk and burden of CVD. The proliferation of wearable technology and the monitoring of sleep quality by smart devices offer promising opportunities for human-centered approaches to sleep optimization (44), intending to improve cardiovascular and overall health and early interventions to prevent the onset of CVD. Creative strategies are still needed to improve long-term adherence to sleep-based lifestyle interventions.

Despite the study's multiple strengths of a nationally representative sample, rigorous study protocol, standard statistical analysis, and cooperation of machine learning methods, some limitations make interpretation more cautious. The study's short follow-up period of 4 years may not be sufficient to analyze CVD events adequately, and a longer follow-up should be considered in further studies. The fact that we only had self-reported sleep duration rather than objectively measured sleep parameters does not allow us to obtain the specific sleep quality of the study population, so additional monitoring of sleep parameters should be considered at follow-up. Self-reported CVD is another limitation. However, multiple studies have shown self-reported CVD data to be reliable (45, 46). In addition, previous studies in other countries have concluded that long sleep duration is also associated with CVD mortality (9, 27). We need to investigate the association of long sleep with CVD in extensive studies of Chinese adults. In conclusion, both sleep duration and Mets are potential risk factors of CVD onset, and short sleep duration increased CVD risk in middle-aged and elderly people with Mets. Adding sleep deprivation to the Mets risk cluster may be a cost-effective and healthy way to prevent the onset of CVD.

## Data availability statement

The original contributions presented in the study are included in the article/supplementary material, further inquiries can be directed to the corresponding author/s.

## Ethics statement

This study was approved by the Biomedical Ethics Committee of Peking University (IRB00001052-11015) and all participants provided written informed consent.

## Author contributions

JS, YS, and YC: study concept and design. YS: acquisition of data. JS and YC: data analysis and interpretation. JS: manuscript writing. YS, YC, and BY: manuscript reviewing editing. Supplementary content, foundation and administrative support: BY and JZ. All authors contributed to the article and approved the submitted version.

## Funding

This research was supported by the National Natural Science Foundation of China (No. 82170316) and the Natural Science Foundation of Hubei Province (2021CFB334).

## Conflict of interest

The authors declare that the research was conducted in the absence of any commercial or financial relationships that could be construed as a potential conflict of interest.

## Publisher's note

All claims expressed in this article are solely those of the authors and do not necessarily represent those of their affiliated organizations, or those of the publisher, the editors and the reviewers. Any product that may be evaluated in this article, or claim that may be made by its manufacturer, is not guaranteed or endorsed by the publisher.

## Footnotes

1 https://charls.charlsdata.com/pages/Data/2011-charls-wave1/zh-cn.html

## Abbreviations

AdaBoost: Adaptive Boosting
BUN: Blood Urea Nitrogen
BMI: Body Mass Index
CVDs: Cardiovascular and Cerebrovascular Diseases
CHD: Coronary Heart Disease
CKD: Chronic Kidney Disease
CRP: C-Reaction Protein
(CHARLS): China Longitudinal Study of Health and Retirement
DNN: Deep Neural Networks
eGFR: Estimated Glomerular Filtration Rate
HbA1c: Glycosylated Hemoglobin
HDL: High-Density Lipoprotein
HF-HRV: Heart Rate Variability
HPA: Hypothalamic-Pituitary-Adrenal
Hb: Hemoglobin
IL-6: Interleukin-6
LR: Logistic Regression
LDL: Low-Density Lipoprotein
MI: Myocardial Infarction
Mets: Metabolic Syndromes
NE: Norepinephrine
SCN: Suprachiasmatic Nucleus
TC: Total Cholesterol
TG: Triglycerides
UA: Uric Acid.
